# Technology to Support Aging in Place: Older Adults’ Perspectives

**DOI:** 10.3390/healthcare7020060

**Published:** 2019-04-10

**Authors:** Shengzhi Wang, Khalisa Bolling, Wenlin Mao, Jennifer Reichstadt, Dilip Jeste, Ho-Cheol Kim, Camille Nebeker

**Affiliations:** 1 The Design Lab, Computer Science and Engineering, Jacobs School of Engineering, UC San Diego, La Jolla, CA 92093, USA; shw198@ucsd.edu; 2 Department of Family Medicine and Public Health, School of Medicine, UC San Diego, La Jolla, CA 92093, USA; kbolling@ucsd.edu; 3 Computer Science and Engineering, Jacobs School of Engineering, UC San Diego, La Jolla, CA 92093, USA; w6mao@ucsd.edu; 4 Division of Geriatrics and Gerontology, Department of Medicine, School of Medicine, UC San Diego, La Jolla, CA 92093, USA; jreichstadt@ucsd.edu; 5 Department of Psychiatry, Sam and Rose Stein Institute for Research on Aging, School of Medicine, UC San Diego, La Jolla, CA 92093, USA; djeste@ucsd.edu; 6 Scalable Knowledge Intelligence, IBM Research-Almaden, San Jose, CA 95120, USA; hckim@us.ibm.com; 7 The Design Lab, Center for Wireless and Population Health Systems, Department of Family Medicine and Public Health, School of Medicine, UC San Diego, La Jolla, CA 92093, USA

**Keywords:** retirement community, co-design, privacy, research ethics, artificial intelligence, robots

## Abstract

The U.S. population over 65 years of age is increasing. Most older adults prefer to age in place, and technologies, including Internet of things (IoT), Ambient/Active Assisted Living (AAL) robots and other artificial intelligence (AI), can support independent living. However, a top-down design process creates mismatches between technologies and older adults’ needs. A user-centered design approach was used to identify older adults’ perspectives regarding AAL and AI technologies and gauge interest in participating in a co-design process. A survey was used to obtain demographic characteristics and assess privacy perspectives. A convenience sample of 31 retirement community residents participated in one of two 90-min focus group sessions. The semi-structured group interview solicited barriers and facilitators to technology adoption, privacy attitudes, and interest in project co-design participation to inform technology development. Focus group sessions were audiotaped and professionally transcribed. Transcripts were reviewed and coded to identify themes and patterns. Descriptive statistics were applied to the quantitative data. Identified barriers to technology use included low technology literacy, including lack of familiarity with terminology, and physical challenges, which can make adoption difficult. Facilitators included an eagerness to learn, interest in co-design, and a desire to understand and control their data. Most participants identified as privacy pragmatics and fundamentalists, indicating that privacy is important to older adults. At the same time, they also reported a willingness to contribute to the design of technologies that would facilitate aging independently. There is a need to increase technology literacy of older adults along with aging literacy of technologists.

## 1. Introduction

The preference of older adults to “age in place,” or to live independently at home, rather than in an assisted living facility, is widely recognized [1,2,3,4]. Individuals who develop disabilities and are no longer able to age in place are likely to be institutionalized in assisted living facilities. These declines, which can occur with advanced age, are key barriers to one’s ability to maintain an independent lifestyle [5,6]. This often leads to more significant mental and physical decline, as well as quality of life decline and increased cost of care, compared to older adults who continue to live independently [7]. As a result, effective means of providing support for older adults are of central public health and ethical significance. In many cases, external aids provide crucially needed assistance that can prolong independent living.

Technologies, such as internet of things (IoT), Ambient/Active Assisted Living (AAL) robots and other artificial intelligence (AI), have been shown to have great potential in fostering independent living, improving mental and physical health, and increasing quality of life [8,9,10,11]. At the same time, they can also reduce caregiver burden, which can lead to more targeted and better quality care [12]. However, despite playing a significant part in successful interventions, adoption of these technologies has been limited [13,14]. One key barrier to wider adoption has been the “top-down” design process that is often used in creating technology for older adults. This process is based on technologists’, or at best geriatricians’, preconceptions of the needs of older adults with little consideration of user perspectives and preferences or their real-world constraints.

While there are a number of studies that have indicated the need for well-designed technologies that meet the needs of older adults [15], few have addressed user-related issues in the design process of these technologies. It has been recognized that effective technologies are those that prioritize the needs and wishes of older adults, general acceptance of potential users, and suitable preconditions for its adoption [16], but this is often difficult to achieve with a top-down design methodology that fails to engage users in the design process. This has frequently created significant mismatches between the needs and preferences of the users and the products that are developed to fulfill their needs. Areas of concern for users include visual appearance, functionality, affordability, platform sustainability, privacy concerns, and interaction complexity [17,18,19]. These mismatches can hinder meaningful adoption and sustained usage, and risk leaving priority needs of end-users unmet. Employment of user- or human-centered design (HCD) involves the end user in the early planning phases to better understand the needs of individuals for whom a product is being developed and to ensure relevant safety, access, and utility are built in [20]. A design process involving end users can reveal untapped areas for improvement, which can lead to improved user satisfaction and lower adoption barriers, and ultimately to much improved support for individuals who wish to age in place [21,22].

The goals of this study were to: (1) involve residents of a local continuing care senior housing community (CCSHC) in conversations about technologies that might facilitate their continued independent living status [23]; (2) assess their privacy attitudes and preferences; and (3) identify whether residents would be interested in co-designing technologies moving forward and if so, how to foster next steps.

## 2. Materials and Methods

### 2.1. Recruitment

Two focus groups were convened at a local retirement community located in San Diego, California, to explore barriers and facilitators to technology adoption as well as interest in participating in a “tech” co-design process. This study was carried out in conjunction with a longitudinal, observational study involving over 100 residents. Both focus groups were held in August 2018. Residents of the retirement community were invited to participate via an IRB-approved flyer. Community leadership helped distribute the flyer and placed copies in the community lobby. Input was solicited from community leadership to determine appropriate time slots for the focus groups. Residents interested in participating were directed to sign up for one of two time slots offered. Selection criteria included any resident of the independent living facility with an expressed interest in the study and willingness to participate in a 90-min group discussion. Residents requiring assisted living or skilled care were excluded from the study. To confirm attendance, an email reminder was sent to those who signed up for a time slot. On the day focus group sessions were held, no individuals were turned away. The study was verified as exempt by the UC San Diego Institutional Review Board. Focus group attendees were provided with an introduction to the project purpose and they gave verbal consent to participate. Each participant received $30.00 as compensation for their participation.

### 2.2. Data Collection

Data were collected via focus group and survey methodology, both of which are described below:

#### 2.2.1. Focus Group Semi-Structured Interview Guide

The focus group protocol was developed with a goal of obtaining the perspectives and guidance of older adults over 65 years old regarding their: (a) use of and interest in technology and connections to personal health; (b) preferences for involvement in participatory design of AI assistive aids; (c) familiarity with terms, concepts, and processes associated with the design of AI aids for aging in place; and (d) advice and preferences for how technology development experts should most effectively communicate such information to enable an authentic and informed participatory design process (see Appendix A). An initial draft of the semi-structured interview protocol was reviewed by a resident leader of the retirement community and revised to incorporate comments.

Focus groups were conducted in a conference room located on-site in the CCSHC’s main building. Each group session was allocated 90 min and was led by a trained focus group facilitator (CN). A student research assistant took notes and managed logistics while two residents volunteered to record individual comments on poster paper (SW). The moderator asked open-ended questions and participants were asked to answer asynchronously. Some questions prompted the participants to reflect on their answers and write down their thoughts on a 3 × 5 card before further questions were asked. Group discussions were digitally recorded (audio) and professionally transcribed. The focus groups aimed to deepen the understanding of the topic as participants built on one another’s discussions and viewpoints. At the end of the focus group session, participants were asked to complete a written survey to gather demographic information as well as to assess privacy attitudes.

#### 2.2.2. Survey

A survey was used to obtain demographic characteristics of the participants with the four scales designed to measure privacy attitudes described below:

##### Westin Privacy Segmentation Index

The Westin Privacy Segmentation Index (PSI) is used to classify participants into three groups: Privacy Fundamentalists, Privacy Unconcerned, and Privacy Pragmatists [24]. The PSI consists of three questions that assess whether consumers trust businesses and regulations to safeguard their privacy.

##### Westin Privacy Concern Index

The Privacy Concern Index (PCI) is another scale that was developed by Westin et al. in the 1990s [24]. Scoring is used to classify participants into three groups: high, medium, and low with respect to the level of privacy concern. The PCI used for this study included five questions from the original six-item version. One item regarding the creation of a privacy commission by the government was omitted as it was unrelated to the present study. The survey also asked participants about their concerns on current threats to their personal privacy. While this question is not part of the PSI or PCI, it was also developed by Westin and Louis, and was used in prior privacy research [24].

##### Internet Users’ Information Privacy Concerns (IUIPC)

The Internet Users’ Information Privacy Concerns (IUIPC) scale by Malhotra et al. consists of ten questions with three subscales (Cronbach’s alpha = 0.92) [25]. The first three questions (1–3) comprise the Control subscale and focus on the extent to which participants believe that control is the key issue with consumer privacy. Questions 4–6 are labeled the *Awareness* subscale and are used to rate the importance of disclosure and awareness of information collection. The last four questions (7–10) are labeled the *Collection* subscale and focus on the amount of information that is collected by companies. Along with these ten questions, two additional questions developed by Malhotra et al. were also included. One asked whether participants falsified their personal information during registration on websites and the other focused on how frequently participants heard about the potential misuse of the information collected from the internet [25].

##### Data Sensitivity

The last section of the survey assessed participant perspectives of the sensitivity of different kinds of personal information. The 12 different types of personal information were rated using a ten-point scale with 1 = “Not sensitive at all” to 10 = “Very sensitive.” The majority of the 12 types of personal information focused on health information (e.g., electronic health records and present fitness), with three types being more general (e.g., internet search terms, GPS).

### 2.3. Data Analysis

The transcribed audio recordings were analyzed using a methodology of “Coding Consensus, Co-occurrence, and Comparison” and rooted in grounded theory (i.e., theory derived from data and then illustrated by characteristics examples of data) [26,27]. Each transcript was independently coded by two project investigators (JR and KB) at a general level in order to condense the data into analyzable units. Segments of transcript were assigned codes based on a priori (i.e., questions in the focus group guide) or emergent themes. In a few instances, the same text segment could be assigned more than one code. The investigators subsequently met to discuss and refine the codes and to develop a final list of codes, construed through consensus, consisting of a numbered list of themes and sub-themes, issues, and opinions. With the final coding structure, interrater reliability was assessed for a subset of 10 pages of each transcript (about half of the total pages), with a kappa value of 0.97, indicating a high degree of concordance between raters.

Quantitative data were analyzed using SPSS.

## 3. Results

Participants included 31 older adults between the ages of 67 and 94 years (mean = 80.0, SD = 6.2) with 20 females and 11 males, and 70% having a college or graduate degree. Most (97%) were White, with 60% reporting an annual income of over $100 K (see Table 1). Two individuals did not complete the demographic and privacy survey.

The qualitative analysis revealed several key barriers toward adoption of technologies and digital platforms, namely: (1) technology usability, (2) technology literacy, (3) data management and privacy, and (4) technology co-design. Below, each theme is defined and characterized by participant comments and survey results.

### 3.1. Technology Usability

The theme of “technology usability” was defined by how accessible a product is to those attempting to use it. Sub-themes point to barriers around user interface making intuitive navigation of a product difficult, and physical challenges that become obvious when technologies are not designed for the older demographic.

#### 3.1.1. Lack of Unified Frameworks and User Interfaces

Because of the fragmentation of different digital platforms and services, there are many cases where the lack of a unified user interface can lead to increased user frustration and compromise usability. For example, one participant was trying to set up an email service and expressed frustration with navigating what should have been a fairly routine task.

“To get email hooked up, to get this done, to get the keyboard… all that stuff, and a lot of folks just give up.”

Another purchased a backup system for her computer, but had no idea how to set it up.

“I’m going 160 days without backup because even the geek group that we have here can’t answer my question in order for me to get it set up.”

Frustration appeared to be a significant barrier, which led to a lack of self-confidence and motivation to pursue using the technology.

“I think technology can, for some people, get to the point where life is more difficult than it was before we had that technology.”

“I have a smart phone. My kids think I can use this, but I don’t really know how to use this, and they bought it for me thinking it would be a great tool, but I don’t really know what to do with it.”

Devices that were easy to use due to having simple features, such as the “on/off” switch for an electronic toothbrush, or plugs that worked regardless of how you inserted it (i.e., the Apple Lightning Connector [28]), were noted as being supportive technologies. Lastly, concerns were voiced about technologies that introduce hazards to older adults, such as the use of power cords. Since having power cords around the house creates a tripping hazard and fall risk, several participants advocated for more wireless functionality.

#### 3.1.2. Increased Mobility and Visual Challenges When Using Technologies

Older adults often face challenges in accessing hardware features or digital content due to a lack of accommodation of their limitations in mobility and decreased visual capacity. In some cases, the technology is designed to keep the battery charge connection out of sight—perhaps for aesthetics or weather proofing. However, when concealed, connectors can be more difficult for an older adult to access. One participant described his experience trying to charge the electric scooter that he uses to get around. Specifically, the connection was underneath the seat, requiring that he either bend over or get on his hands and knees to locate and connect the plug for charging.

“One of the biggest frustrations that I’ve seen was folks with power carts and a lot of them, to charge the battery, the plug is way down underneath your seat…. Couldn’t bend over to put it in, can’t see it…”

There are also times when a simple solution, such as replacing a battery, proves challenging as evidenced by this comment:

“I have had more calls from people who say, “I just put a brand-new battery and it doesn’t work.” They put the battery in backwards (laughter) and, at times, it burns out the unit.”

Participants noted that the difficulty in replacing a battery was related to the inability for many to visually see the positive and negative symbols.

#### 3.1.3. Recommendations for Improving Technology Usability

Participants were pragmatic in their recommendations for improving usability. Simple instructions, fewer buttons, larger fonts, and speech-activated tools were noted during the discussion.

“Why don’t they have a senior version or an app that can get to on, off, volume up/down, channel, and make it kind of simple?”

Of interest, but not surprising, were also suggestions such as having a universal remote to operate the television and peripheral devices—technologies that are often already on the market as finished products, but plagued by a lack of awareness of their existence.

### 3.2. Technology Literacy

Technology literacy is a theme defined by having sufficient knowledge to independently understand the instructions to facilitate use of a technology. Sub-themes point to knowledge barriers, need for resources, and data management.

#### 3.2.1. Knowledge Barriers

A lack of understanding of modern technologies and digital platforms was identified as a barrier resulting in underutilization of technology and dependence on others to operate basic features. Participants mentioned purchasing services (e.g., Netflix) they did not use, because they could not understand how it worked.

“I know I’m looking for this connection, but I don’t know what it’s called, I don’t know what the things are, and so there is no … terminology, you know … um.”

Another participant commented that in order to use her smart phone, she needed guidance from her granddaughter. Moreover, many of the participants left the workforce before technology was integrated into the daily work flows in a significant way, leaving them without the vocabulary or basic skills needed to function in the digital age. One participant described this tech literacy gap as follows:

“…I retired 20-something years ago, so I didn’t have the opportunity to work with them [technology] at work. So we got less work-based training on them and I don’t understand the language of it. Trying to hook a printer up to my laptop—they said to put in the IPP [sic IP] address. Uhh, I can’t find it… you know, and things like that, I don’t know what they are talking about.”

#### 3.2.2. Recommendations for Improving Technology Literacy: Need for Resources

The tech literacy problem could be addressed with the “how to” manuals that accompany technology devices; however, the “Getting Started” instructions were described by participants as too technical due to the unfamiliar terminologies that were used. Most people relied on family members to help with setup, but this did not always result in the type of help they needed.

“Show me, slow down, and it’s hard to get ‘em to slow down. And you know, and I feel like I’m being a burden or they just don’t think Nana is smart enough. Maybe I’m not, but I could try to be if they were a little more patient.”

One participant asked if university students were being trained to help older adults learn to use the technologies. From a technology perspective, user interface is optimal if fewer, rather than more, support personnel are needed. The fact that older adults need assistance in using technologies is indicative of suboptimal designs for this demographic.

### 3.3. Data Management and Privacy

The theme of data management and privacy is characterized by three sub-themes: (1) how data can be collected and used; (2) whether knowledge gained is shared in a form that results in value to the participant; and (3) privacy attitudes.

#### 3.3.1. Data Collection and Use

While highly educated, most participants lacked understanding of the granularity of data that can be captured with pervasive sensing technology and the associated analytics used by digital platforms to identify patterns. The mystery of AI, including what it is and how it works, contributed to fears of data loss or being harmed from decisions made from their personal data.

“If they’re [the technology] so sensitive, they know three weeks before we know what’s going wrong with our bodies. It seems to me that that kind of information could really be compromised, and seniors could, uh, who are very vulnerable, could really be hoodwinked more easily.”

#### 3.3.2. Return of Value

Moreover, the idea that data could be collected about them without a return of value was problematic. Participants expressed a desire for more instantaneous and understandable feedback, especially when participating in health research. The lack of feedback could potentially hamper enthusiasm for research study participation.

“You need to talk to your doctor about X, Y and Z. Um, but if you just keep gathering data and nothing happens to that data … um … except that you can look at it and … and you can’t really interpret it…”

#### 3.3.3. Privacy

There was widespread desire by participants to understand how to use different technologies and how to control personal data. In addition, in order to better understand participants’ privacy attitudes, this issue was discussed during the focus group, and participants’ attitudes were measured via a survey.

##### Westin Privacy Concern Index

For this index, three questions are used to classify a person as low, medium or high with respect to their concern about privacy in the context of trust that business and law will protect their privacy.

**Figure 1:**
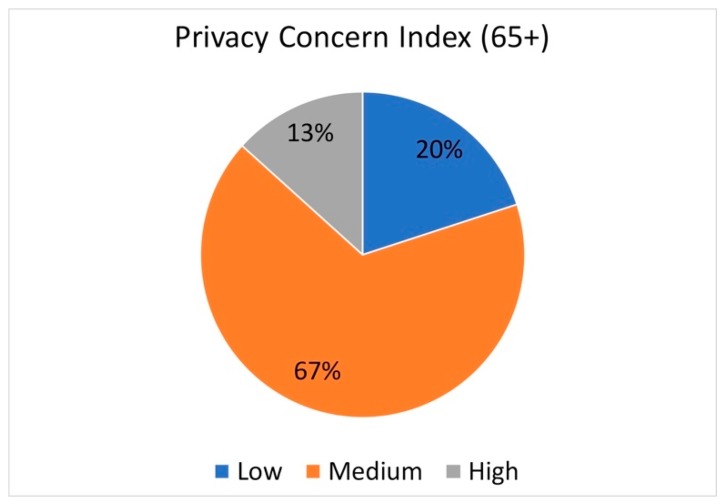
Westin Privacy Concern Index Results.

A majority of participants (66.7%) reported a medium privacy concern compared with 20% reporting a low concern, and 13.3% reporting a high concern (see Figure 1).

##### Westin Privacy Segmentation Index

**Figure 2:**
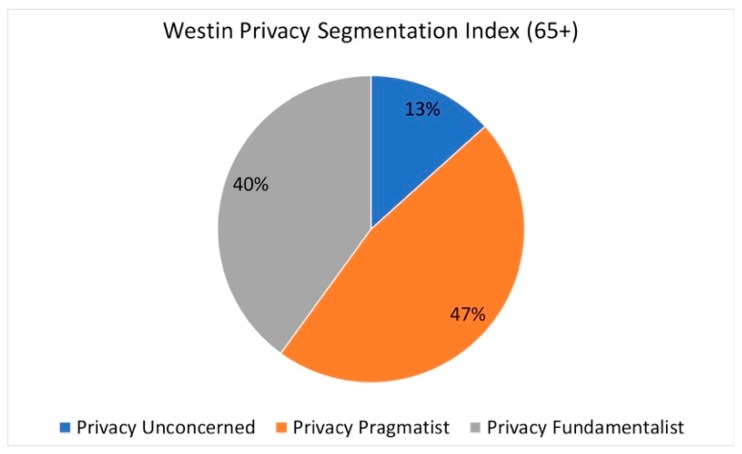
Westin Privacy Segmentation Index Results.

Between 1979 and 2001, Westin randomly selected U.S. citizens to gauge privacy attitudes across a variety of domains, including health information, consumer and e-commerce [29], and identified three key privacy categories: pragmatists, fundamentalists or unconcerned [24]. Results from Westin’s “Privacy On and Off the Internet” survey revealed that 25% of those surveyed were fundamentalists, 55% were pragmatists, and 20% were unconcerned [24,30]. Fundamentalists were described as having a high value for privacy, believing they own their information, and supporting strong laws and regulations to secure privacy rights. Pragmatists were characterized as open to information disclosure if to a trusted entity providing a personal benefit; and unconcerned were described as not having a high need for privacy and control of information [30]. While there has been some criticism of Westin’s scale, it is a potentially useful baseline for understanding privacy attitudes. For the purpose of this study, we used this scale to compare our sample with national survey results. Nearly half of our older adult participants (46.7%) were categorized as “privacy pragmatist”, compared to 55% from Westin’s sample. Only 13% of our older adult sample was considered “privacy unconcerned”, with 40% categorized as “privacy fundamentalist”, compared with 20% and 25% of Westin’s sample, respectively (see Figure 2). When asked about the level of concern regarding threats to personal privacy in America nowadays, a majority of the participants (58.1%) reported being “somewhat concerned”, with 29.0% being “very concerned”. Compared to national averages, our sample of older adults scored lower in the privacy pragmatic and unconcerned categories and much higher in the privacy fundamentalist category.

Results of the Westin Privacy Concern Index showed that a majority of older adults in our sample had a medium or high privacy concern (80%) with 40% categorized as privacy fundamentalist using the Privacy Segmentation Index. These results indicate that the older adults we sampled are less willing to share information about themselves with others. However, we learned during the focus group discussion that participants were willing to share information if they received something in return, which is more aligned with the privacy fundamentalist classification where people weigh sharing information based on what they get back. For example, with respect to sharing personal information, one participant stated:

“That’s fine, you can take all the data you want, I mean … but is it gonna be of benefit to me?”

Another participant liked the idea of getting personalized feedback from artificial intelligence tools as noted here:

“Well, I think if you can get some sort of readout that is, you know, available from the unit in your apartment, the status of where you are today, to be interactive in a sense, broadcasting the information that is … is collected about you and be analyzed by the artificial intelligence obviously to give you some kind of status, you know, you … you’re doing okay today or … or you ate too much yesterday.”

##### Internet Users’ Information Privacy Concerns (IUIPC):

**Figure 3:**
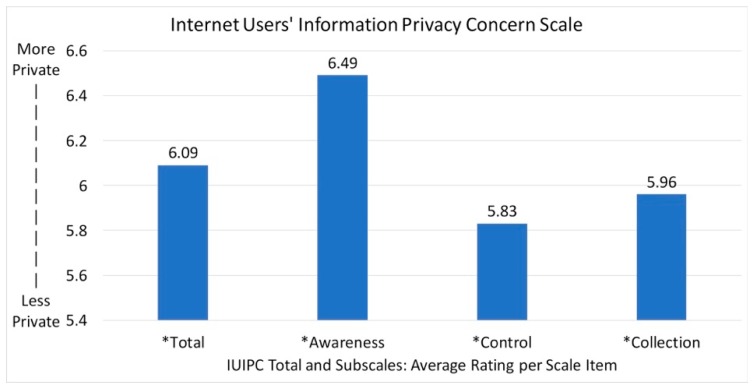
Internet Users’ Information Privacy Concerns (IUIPC) Results.

The IUIPC is a 10-item scale with a high internal consistency (Cronbach’s alpha = 0.90). The level of internet privacy concerns was high among participants with an average rating of 6.1 out of 7 (SD = 1.3). The *awareness* subscale score was high with an average rate of 6.5 out of 7 (SD = 1.2). This subscale showed that 70% of the older adults were aware of the issue of personal information collection online and strongly agreed that disclosure of information usage was important. Additionally, the *control* subscale was moderate with an average rate of 5.8 out of 7 (SD = 1.6). In fact, a majority (50%) of the older adults strongly agreed with the idea that control is the key issue with consumer privacy. These older adults also reported a moderate level of concern on the *collection* subscale with an average rate of 6.0 out of 7 (SD = 1.6). Specifically, 60% of participants felt offended about the amount of information that is being collected by companies (see Figure 3).

When asked about the percentage of time older adults falsify their personal information during registration on a website, 86.7% of the older adults reported either never falsifying their personal information or falsifying their information less than 25% of the time. This suggests that older adults are less likely to take certain online privacy protection methods to protect themselves. When asked about how frequently they heard about the potential misuse of the information collected from the internet, the mean score was 4.5 (SD = 1.6) out of 7 (Very Much), indicating that most of the older adults had heard or read some information on this topic.

##### Sensitivity to Personal Information

**Figure 4:**
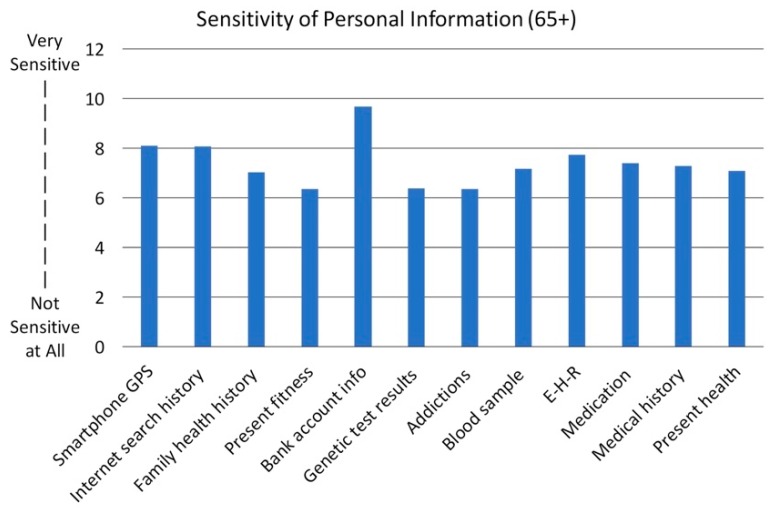
Sensitivity of Personal Information Analysis.

To better understand privacy attitudes, our survey asked the participants to rate the sensitivity of 12 different types of personal information (see Figure 4). The results indicate that participants regarded their bank account information as the most sensitive data type with an average sensitivity rate of 9.7 (SD = 1.1) out of 10 (Very Sensitive). Across all 12 sensitive information types, participants rated present fitness and addictions as having the lowest sensitivity, with an average rating of 6.4 (SD = 3.0 and SD = 4.0, respectively) out of 10 for both. Next to bank account information, smartphone GPS data and internet search history ranked among the most sensitive types of data, both with an average rating of 8.1 (SD = 2.8) out of 10, suggesting that older adults generally consider online information as more sensitive. The highest sensitivity rating on health information was the electronic health records (EHR) with an average sensitivity rating of 7.7 (SD = 3.4) out of 10.

### 3.4. Co-Design of Technology

Participants favored the idea of participatory design and were eager to participate in a co-design process. The consensus was that as people who have lived experiences being older, they brought a perspective to the tech development process that might not be present otherwise.

“… ethics and morality and seeing further from having lived longer that I think collective wisdom of the elderly might be extremely important in the checks and balances put in place.”

“I think it’s a deal of bioethics. Sometimes the 85 and above have more human knowledge than the people working in the industry.”

Participants also expressed ideas around what an ideal design project might include to better meet their needs.

“Why can’t there be a feature on the TV that I can get the sound to come straight to my hearing aid electronically?”

“If they would come up with a universal remote that worked for the TV, for the iPhone … for everything so that you could program into it that you could remotely operate… I think that would solve some of the problems we all have.”

Clearly, there is a significant desire to participate and contribute to the ideation and development process, which could lead to technology better designed for adoption by older adults.

## 4. Discussion

The growth of general purpose and healthcare-related technologies has created the potential to help more older adults to age in place. Living independently is preferred by older adults and smart technologies like IoT, AI, and AAL can provide the necessary assistance. Due to improvements in communication and remote data gathering capabilities by healthcare providers and researchers, operationalizing smart communities will become more dependent than ever on sensors and predictive analytics of collected data. The results from this study reveal barriers to the adoption of technologies and facilitators that could foster increased access to and usability of technologies to support independent living. Factors identified through this study were: (1) technology usability, (2) technology literacy, (3) data management, (4) privacy attitudes, and (5) co-design.

### 4.1. Technology Usability

Older adults in our study tended to associate adoption of new technologies with a lack of confidence in their ability to understand or access them. A significant source of frustration in their interactions with digital products lay in the inadequacies in software and hardware interfaces that permit access to different functionalities. Participants gave examples of technologies they interacted with every day and identified specific examples of problems with their access to these technologies.

The physical decline that can occur as people age creates physical access barriers in technologies. These can be attributed to the dimensions and locations of certain components and how they interface with power sources and other technologies to conduct data transfer or data input. Another noticeable concern in physical access is the existence, or lack thereof, of visibility enhancement features. Visual aids are often inadequate or poorly designed for common use cases that can allow for easier access to content displayed in a visual medium.

It has been suggested that a key motivation in technology adoption by older adults is the presence of a significant perceived benefit [31]. Despite the fact that many participants in the study indicated ownership of a diverse set of modern devices, many of the features that participants wished for in future technologies already existed on the personal devices they already possessed. The lack of knowledge of the existence of these functionalities can vastly diminish the perceived potential of many technologies, affecting adoption or continual usage.

### 4.2. Software Interface

The software interface is also a potential source of friction for older adults. Lack of familiarity with and understanding of technology can make it difficult for older adults to be at ease while operating user interfaces. Because older adults were not introduced to modern digital work environments until later in life (or in many cases not at all), their ability to adapt to changes is hindered by a lack of fundamental knowledge in how digital infrastructures operate and how data is utilized.

While the lack of understanding in operating a device could be alleviated somewhat by instructional material documenting steps to access functionalities, older adults tend to rely on static content, such as printed manuals, to fulfill this need. Few participants in the study were capable of effectively utilizing online instructional and troubleshooting materials. In some cases, the barrier was a small font size that was difficult to read, even with prescription glasses. The vocabulary was often unfamiliar (e.g., Bluetooth) and proved meaningless when trying to understand instructions. For many, this meant an increased reliance on assistance from younger and more “tech savvy” family members. While relying on family is a possible solution for those who are fortunate enough to have younger and helpful family members who are more knowledgeable, it is less effective in helping older adults solve future problems.

Modern software and internet platforms have also adopted the model of constant incremental updates and iterations to adapt to user preferences. This has created fluid interfaces that change without warning, quickly rendering previous usability knowledge and documentation obsolete. This unpredictability is especially problematic with the fragmentation in interface philosophies on different digital platforms, necessitating repeated familiarization processes to keep pace with the latest changes. How to make these incremental updates while considering the impact on the digital novices is important if we are to design for needs of an older demographic.

### 4.3. Data Control and Privacy

A large majority (87.1%) of the participants indicated that they were concerned with data privacy in their day-to-day usage of technology. This high level of concern with controlling their personal data suggests a hesitancy in adopting a technology or submitting personal data to a digital platform. This mistrust and misunderstanding of the handling of data can be an especially serious obstacle to the adoption of technology that requires large amounts of personal data to be effective, such as machine learning algorithms.

Another concern voiced by the participants was a lack of feedback from data collected by digital devices or researchers. The awareness subscale in IUIPC showed that 70% of the participants were aware of the online information collection and strongly agreed that disclosure of information usage is important. However, the lack of feedback could potentially lead to decreased enthusiasm to participate in studies or an unwillingness to provide personal data. This is particularly an area of interest for digital health research, where the data collected and their analysis can be of high interest to the participants who are concerned about their health. At the same time, it is often unclear how to return the data back to the research participants in a manner that is meaningful to them. In traditional clinical research, research data are rarely returned to the participants. As such, there is a lack of a clear pathway to determine what would be meaningful (e.g., raw data or a short report) nor how often to provide feedback. The answer is likely to be person-specific—for instance, a person who requests and can process complex information versus someone who is satisfied with a very brief summary. In keeping with the growing focus on personalized medicine, there should be a framework for providing personalized data feedback.

### 4.4. Implications for Creating Age-Friendly Communities

As the number of older adults increases, the World Health Organization has initiated a movement to establish age-friendly communities [32]. An important component of this initiative should be identifying technologies that support aging in place. Our early stage HCD research sheds light on important issues that are unique to older adults specific to privacy and technology literacy. Engagement of older adults in the design of technologies is often overlooked or an afterthought. Technologies that are commonly used by older adults are often developed without consulting them at the early stage of product conception. This top-down design model means that user input is only received by the product developer after it is completed, making it much harder to alter in order to fit user needs. Our study showed that older adults are experts in their lived experiences and can identify the potential barriers to technology adoption and use.

In this study, participants voiced their concerns about technologies they interacted with daily, albeit with varying levels of success, and offered ideas for how to improve these products. One issue was their lack of understanding of fundamental technology concepts. A common barrier to the participatory design process involving older adults is the lack of expertise in product development and programming [33]. Because of this technology literacy gap, there is significant potential value in providing an educational component in the co-design process to overcome this issue. While impractical to educate older adults on more complicated topics in computer science and human computer interaction, basic knowledge about current technologies and how they interact with each other would be immensely valuable. For instance, one participant commented that the facility personnel spend a lot of time letting people into their apartments because residents often misplace or forget their keys. An eye scanning or finger print sensor that could be used to unlock the door of the residence, or a system that mimics the proximity-based keyless lock system on modern cars, was suggested by a participant. By gaining a high-level understanding, the resulting ideas and concepts generated by older adults can be more meaningful, particularly in the prototyping stage of the participatory design process, where practical knowledge is needed [34].

In addition to the understandability and usability of different technologies, concerns were raised by participants over the use of data and the importance of privacy and control. This feedback is especially useful when designing technologies for older adults, who may have a very different perception of data and expectations around privacy than younger generations. Many indicated their willingness to provide more sensitive data if it meant getting meaningful feedback on the status of their heath. At the same time, they were also reluctant about sharing data of other categories due to hacking or data loss concerns. This indicates the importance of addressing privacy concerns in different scenarios for different technologies. A participatory design process that values privacy could be a key factor in improving user adoption.

By including residents in this formative research, we were able to identify what would be needed to engage older adults in the design process in a meaningful way and what they would like to receive in the form of feedback. With a better understanding of the technology that they are using, older adults can shape the design philosophy to better serve their needs as users. A next step in this research is to develop a co-design process that incorporates technology education as a component with a goal of increasing “tech literacy.” We anticipate this education will facilitate identifying and prioritizing problems that can be addressed with a technological solution that residents help to co-design.

### 4.5. Limitations

The results presented here are part of a larger study to determine how AI can be used to assess individual cognitive and physical status through the use of traditional means and sensor technologies. Due to the parameters of the larger study, the sample drawn for this study involved people residing in a CCSHC, which is not a random nor representative sample of older adults. These results are also based on a relatively small sample of 31 participants. However, all participants contributed to the discussion and a data saturation point was reached. Finally, the PSI, PCI and IUIPC scales were developed for testing consumer and internet privacy.

## 5. Conclusions

This study demonstrates the significant gap that exists between the potential benefits offered by technologies such as AI and other AAI and the barriers that plague older adults in the adoption of these technologies. Education is critical not only for older adults, but also for technologists. While increasing “technology literacy” of older adults can provide meaningful improvements in helping these users interact more successfully with technology, we also must address the need to educate technology creators about older adults—i.e., increasing “aging literacy” of technologists. This education can occur through pragmatic exercises that involve partnering with older adults to design future technologies. Through co-design partnerships, we can create technologies that are useful and capable of reducing barriers at the design phase. Rather than intervening after a product is in the market place, we can preempt the problems introduced by low technology literacy and fundamentalist privacy attitudes. Moreover, feedback loops can be built in that will help older adults to better understand their data and how these data are used to predict their healthcare needs.

## Figures and Tables

**Table 1:** Demographics of the Sample.

**Age (mean, SD)**	80.0 (6.2)
**Gender (n, % female)**	20 (64.5%)
**Highest education (n, %)**	
Graduated from high school or GED completed	4 (13.3%)
Graduated from 2-year college	5 (16.7%)
Graduated from 4-year college	6 (20.0%)
Completed some post-college education	6 (20.0%)
Completed Master’s degree	6 (20.0%)
Completed professional degree or Ph.D.	3 (10.0%)
**Ethnicity (n, %)**	
Hispanic or Latino	2 (6.5%)
Not Hispanic or Latino	28 (90.3%)
NA	1 (3.2%)
**Race (n, %)**	
Caucasian/White	30 (96.8%)
Asian	1 (3.2%)
**Approximate annual household income (n, %)**	
$50,000–$99,999	12 (40.0%)
$100,000–$149,999	14 (46.7%)
$150,000–$199,999	3 (10.0%)
$300,000 or more	1 (3.3%)

**Figure A1:**
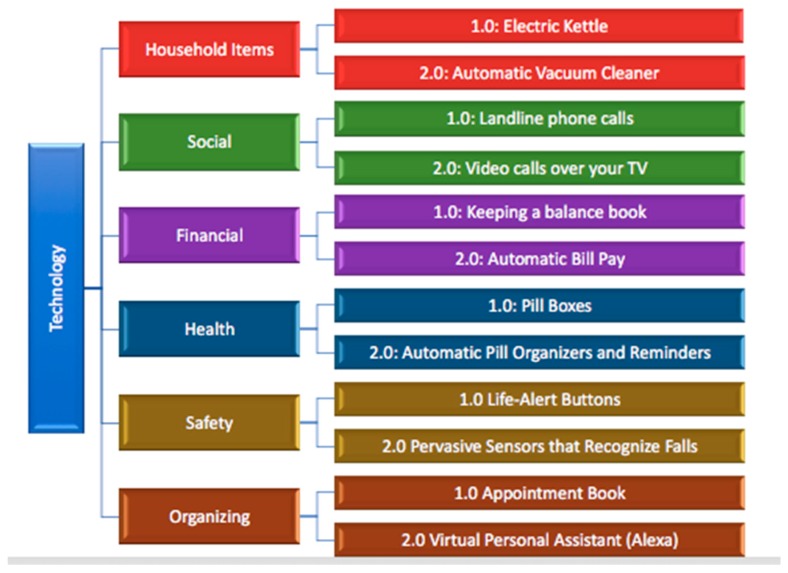
Technology by Domains.

## Appendix A

Focus group protocol

CO-DESIGN TECHNOLOGY FOR AGING IN PLACE—HEALTHY AGING

August 9, 2018

### INTRODUCTION

**Welcome**: Hello everyone! I’d like to thank you for accepting our invitation to participate in this focus group.
**Confidentiality**: Your contributions to our discussion are important and Shengzhi will be taking notes to help us remember what we discuss today. To make sure we don’t forget anything, we are audio recording each session. In our transcript of the recording, we will not identify you by name and your responses will be confidential. Only members of our research team will have access to the recording, transcripts and our notes.
**Purpose**: We are conducting this research as part of a UC San Diego Health Sciences project on technology-enabled health research. We’ve asked you here today to talk about how technology might be used to facilitate living independently. We are also interested in knowing how your think about your health information and privacy. In addition to asking you to respond to questions, I will also ask that you complete a survey which will take about 10 min.
**Consent:** As with any research, you are free to ask questions at any time and your involvement is completely voluntary. If you have any concerns about being recorded or decide you don’t want to participate in this focus group, please know that you can leave at any time and there will be no hard feelings. To acknowledge your time and contributions, we will give you a $30.00 script that can be exchanged for cash at a local bank.
So, to get started, I would like mention some basic instructions for how this works…
  This session will last about 90-min. Near the end of our time we will ask you to complete a survey. After that, we will give you a check that’s called “script” that you can take to the bank to exchange for cash.
  We are interested in your thoughts and opinions—there are no right or wrong answers here. We expect that there will be some areas where most people agree but, there will also be times when there is disagreement. That’s not a problem—we want to be respectful of each other’s opinions. Differences of opinion are expected so feel free to share yours even if it’s different.
  Please make sure everyone has a chance to chime in and please don’t interrupt each other!

A. **Curiosity about technology and connection to personal health**
  1. **Digital technologies you currently use**
    There is growing interest in how digital technologies can be used to support healthy living and aging-in-place. To get started, I would like you to think about what “digital technologies” you use in your daily life.
    Feel free to use the 3 × 5 cards in front of you to jot down any thoughts you have.
    *<Give 1-2 min to think>*
    Okay, what are some of the technologies that you’re using?
    *<Write key words identified by participants on butcher paper>*
    Think back to a time when you felt that you’d like to use a technology like a smart phone app or Facebook and were not quite sure how to do it? Can you tell us about that?
    Think back to a time when you felt that using a technology or a smart phone app has made you feel great. Can you tell us about that?
  2. **Technology use over time**
    We’ve talked a little about how we think about technology—we’re also curious about how your thoughts about technology has change over time.
    Is the way you think about using technology different than how your family might think about it?
    Why did you start using the technologies on your list? Why have you continued using them?
  3. **Health Practices and Tools**
    Now, we want to talk about how you keep yourself healthy.
    Think about the kinds of tools you use daily to track your health and to keep yourself healthy.
    Write those down on the index card.
    *(Examples: hearing aids, blood sugar or blood pressure monitor, scale, exercise machines, fitness trackers, pill trackers (whether a box or a digital device), medication reminders, mail-order prescription delivery services, safe-alert buttons, diet/nutrition aids, electronic health record monitoring, canes, wheelchairs, etc.)*
    Why do you use these tools or adopt these practices? How did you come to acquire them?
    Why do you keep using them?
    Think of a time that using your health practice or tool was frustrating, or when you struggled to use it correctly. Can you tell us about that?
    Think of a time that your health practices or tools has made you feel reassured, or safer. Can you tell us about that?
    How could keeping track of your health and keeping yourself healthy be made easier?
    What would make you feel confident transitioning adopting a new health practice or tool?
  4. **Health Data and Privacy**
    With technology, there have been problems with handling of personal information and people are worried about their privacy.
    How would you describe the characteristics of a very private person?
    How about a not at all private person? *Make notes on your 3 × 5 cards*
    Do you think privacy means something different today than say 30 years ago?
    Is the way you think about privacy different than how your family might think about it?
    Most people think about their health information being located in their electronic health record but, nowadays, our information is found on Facebook, Twitter as well as in sensor technologies and apps that we download onto our phones.
    There are now fitness devices and apps that can track your steps, diet and sleep as well as mood.
    Do you currently use any apps or wearable devices?
    What are you using and what do you like or dislike about it?
    These devices capture information that could be personal and health related.
    Would you want to control who has access to this information? Why or Why Not?
    Would you want to share information from your personal devices or apps with your doctor?
    What about with researchers like me?
    We are interested in using wearable sensors to learn about how people live in their everyday lives.
    *Show a variety of devices (lumo, empatica, sensecam, authographer, garmin, fitbit)*
    Wearable Sensors
    What do you think about the wearable camera? Would you wear it for a day or a week? What concerns would you have? What would motivate you to wear it?
    What do you think about the wrist worn devices? Would you wear it for a day or a week? What concerns would you have? What would motivate you to wear it?
    What do you think about the waist worn devices? Would you wear it for a day or a week? What concerns would you have? What would motivate you to wear it?
    What do you think of the overall design of these devices? Can you get it on/off easily? Is the information that is collected useful to you?
    Home Sensors
    What do you think about having sensors placed in your apartment that could alert you or a friend about how you’re doing?
  *Show examples of data produced.*
B. **Preferences for involvement in participatory design of intelligent assistive technologies**
  1. **Interest in co-design**
    Some researchers are trying to make activities such as tracking your health, keeping you safe, taking care of your home, communicating with your family even easier with new technologies and tools. *< Show figure of technologies >*
    Now, researchers, and especially engineers and technology developers, might think they know how to best help you, but they also need your input to make sure that they create products that actually fulfill *your* goals and are easy for *you* to use.
    Would you be interested in having conversations with technology makers to guide the design process of products that are specifically geared toward people in retirement communities?
    If you could develop technology to improve your life in any way, what would you develop?
    Do you have an idea of what it would look like? Could you draw a picture of it?
  2. **Familiarity with terms, concepts, and processes associated with the design of assistive technologies for aging in place**
    **Term 1: Aging in Place**
    What does “aging in place” mean to you?
    What kinds of technologies do you think could support aging in place?
    What would “success” mean for the design of aids for aging in place?
    **Term 2: Participatory Design**
    Participatory design means that stakeholders are involved in the design of new products and devices. What do you think this means in practice?
    What can you contribute?
    How would you think of your role on the design team?
    What type of process would you want to feel like you a part of it?
    **Term 3: Assistive Technologies/Intelligent Assistive Technologies**
    *<Show videos or prototypes, such as: https://www.smithsonianmag.com/innovation/how-will-artificial-intelligence-help-aging-180962682/>*
  3. **Advice and preferences for how technology development experts should most effectively communicate such information to enable an authentic and informed participatory design process**
    We want to know how we could maximize your involvement in a participatory design process.
    How much time would you want to spend?
    Do you want to do it at your home, or do you want to meet elsewhere?
    How often can you fit this in?
    Would you be willing to test out devices while they are still in development? Why or why not?
    That’s the end of our session. Thank you for participating!
